# Supplementary material for: An open-source framework for large-scale, flexible evaluation of biomedical text mining systems
Source: J Biomed Discov Collab. 2008 Jan 29;3:1. doi: 10.1186/1747-5333-3-1 (PMC2276192; doi:10.1186/1747-5333-3-1)
Supplement: Additional file 1 — Evaluation platform source code. The additional file contains the software evaluation framework discussed in this paper. The most current release can be downloaded from SourceForge.net [1]. See the accompanying README file for instructions on installation and use. Also included in the distribution is a collection of UIMA wrappers for some commonly used BioNLP tools and annotated corpora. [file 1747-5333-3-1-S1.zip › bionlp_uima_repository-1.0.1/docs/api/src-html/edu/uchsc/ccp/util/external/penn/PennBioTokenizer_Util.html]

```
001    package edu.uchsc.ccp.util.external.penn;
002    
003    import java.util.ArrayList;
004    import java.util.List;
005    
006    import edu.uchsc.ccp.util.nlp.GenericTextUtils;
007    import edu.uchsc.ccp.util.nlp.annotation.Annotator;
008    import edu.uchsc.ccp.util.nlp.annotation.TextAnnotation;
009    import edu.uchsc.ccp.util.nlp.mention.ClassMentionTypes;
010    import edu.uchsc.ccp.util.nlp.tokenizer.Tokenizer;
011    import edu.upenn.cis.tokenizers.BioTokenizer;
012    
013    public class PennBioTokenizer_Util implements Tokenizer {
014        private BioTokenizer bt;
015    
016        private Annotator annotator;
017    
018        public PennBioTokenizer_Util(String tokenizerModelFile) {
019            /* Initialize a new BioTokenizer */
020            bt = new BioTokenizer(tokenizerModelFile);
021            /* Initialize a new Annotator */
022            annotator = new Annotator(new Integer(-1), "", "PennBioTokenizer", "PENN");
023        }
024    
025        /**
026         * return a list of TextAnnotations. One for each token.
027         */
028        public List<TextAnnotation> getTokensFromString(String inputText, String inputDocumentID) {
029            String tokenizedText = bt.tokenize(inputText);
030            String[] toks = tokenizedText.split(" ");
031    
032            List<TextAnnotation> tokenAnnotations = new ArrayList<TextAnnotation>();
033    
034            int startLookingForTokenAtIndex = 0;
035            for (String tok : toks) {
036                
037                List<TextAnnotation> tokens = GenericTextUtils.findTargetInText(tok, inputText, startLookingForTokenAtIndex, inputDocumentID,
038                        ClassMentionTypes.TOKEN, annotator, true);
039                if (tokens.size() == 0) {
040                    /*
041                     * could be that tokenizer cut through the middle of a word which it seems to do, so get the last token that was found,
042                     * append it to the current one and search again
043                     */
044                    tok = tokenAnnotations.get(tokenAnnotations.size() - 1).getCoveredText() + tok;
045                    startLookingForTokenAtIndex = tokenAnnotations.get(tokenAnnotations.size() - 1).getAnnotationSpanStart() - 1;
046                    tokenAnnotations.remove(tokenAnnotations.size() - 1);
047                    tokens = GenericTextUtils.findTargetInText(tok, inputText, startLookingForTokenAtIndex, inputDocumentID,
048                            ClassMentionTypes.TOKEN, annotator, true);
049                    if (tokens.size() == 0) {
050                        /*
051                         * try it once more... could be that tokenizer cut through the middle of a word which it seems to do, so get the last
052                         * token that was found, append it to the current one and search again
053                         */
054                        tok = tokenAnnotations.get(tokenAnnotations.size() - 1).getCoveredText() + tok;
055                        startLookingForTokenAtIndex = tokenAnnotations.get(tokenAnnotations.size() - 1).getAnnotationSpanStart() - 1;
056                        tokenAnnotations.remove(tokenAnnotations.size() - 1);
057                        tokens = GenericTextUtils.findTargetInText(tok, inputText, startLookingForTokenAtIndex, inputDocumentID,
058                                ClassMentionTypes.TOKEN, annotator, true);
059                        if (tokens.size() == 0) {
060                            System.err.println("PENN BIOTOKENIZER --- COULD NOT FIND TOKEN: " + tok);
061                        } else {
062                            tokenAnnotations.add(tokens.get(0));
063                            startLookingForTokenAtIndex = tokens.get(0).getAnnotationSpanEnd() - 1;
064                        }
065                    } else {
066                        tokenAnnotations.add(tokens.get(0));
067                        startLookingForTokenAtIndex = tokens.get(0).getAnnotationSpanEnd() - 1;
068                    }
069                } else {
070                    tokenAnnotations.add(tokens.get(0));
071                    startLookingForTokenAtIndex = tokens.get(0).getAnnotationSpanEnd() - 1;
072                }
073            }
074    
075            return tokenAnnotations;
076        }
077    
078    }
```
